# Estimating future variant Creutzfeldt-Jakob disease cases in the UK: a cohort-based probabilistic model

**DOI:** 10.1016/j.lanepe.2025.101502

**Published:** 2025-11-10

**Authors:** Barnaby Roberts, James Riley, Thomas J. Evans, Richard Knight, Jean Manson, Graham Medley, Manisha Upadhyay, Katy Sinka, Simon Mead

**Affiliations:** a Department of Health and Social Care, 39 Victoria Street, London, SW1H 0EU, UK; b School of Infection and Immunity, University of Glasgow, Glasgow, G12 8TA, UK; c Centre for Clinical Brain Sciences, University of Edinburgh, Edinburgh, EH16 4SB, UK; d Emerita Chair of Neurodegenerative Diseases, University of Edinburgh, Edinburgh, EH16 4SB, UK; e London School of Hygiene and Tropical Medicine, Keppel Street, London, WC1E 7HT, UK; f UK Health Security Agency (UKHSA), 61 Colindale Avenue, London, NW9 5EQ, UK; g MRC Prion Unit at UCL, Institute of Prion Diseases, 33 Cleveland St, London, W1W 7FF, UK

**Keywords:** CJD, Variant CJD, BSE, Modelling, Public health

## Abstract

**Background:**

Variant Creutzfeldt–Jakob disease (vCJD) is a fatal prion disorder linked to dietary exposure to bovine spongiform encephalopathy (BSE). The epidemic peaked in the early 2000s, and no new cases have been reported in the UK since 2016. However, uncertainties remain regarding potential future cases, particularly in individuals with non-MM prion protein gene codon 129 genotypes, and possible secondary transmission via blood transfusion. We aimed to update risk estimates with recent data, informed by a probabilistic modelling approach.

**Methods:**

We developed a cohort-based probabilistic model for variant CJD incidence incorporating genotype-specific attack rates and incubation periods, accounting for competing mortality risks. Model parameters were calibrated using historical case data and life-table analyses. We explored multiple scenarios, including sensitivity analyses with alternative incubation period distributions and potential missed diagnoses. Secondary transmission risk was assessed using historical transfusion-linked cases and epidemiological data.

**Findings:**

In the base-case scenario, our model estimates a 48% probability that no further vCJD cases will occur, with a mean forecast of 2.7 additional cases. Allowing for missed past cases, estimates increased to a mean of 3.0 cases (one missed case) and 4.9 cases (four missed cases). Sensitivity analysis using a Cauchy incubation period distribution rather than a log-normal distribution increased estimates to 6.6, 9.4, and 17.9 cases, respectively. A plausible worse-case scenario, assuming very long incubation times and higher susceptibility in non-MM individuals, projected up to 65 cases of vCJD over coming decades, peaking in the 2030s. Secondary transmission risk remains negligible given transfusion safety measures, including leukodepletion since 1999.

**Interpretation:**

Our findings suggest that vCJD is unlikely to re-emerge at significant levels. However, ongoing case ascertainment and scrutiny remains critical due to uncertainties in non-MM incubation and potentially, the emergence of previously unrecognised prion strains. Continued neuropathological case investigation, maintenance of core blood safety policies, and periodic risk reassessments are essential to ensure public health preparedness and avoid unnecessary restrictions.

**Funding:**

No specific funding other than salaries of the authors.

**Research in context**

**Evidence before this study**

We searched PubMed for primary epidemiology, modelling, transfusion transmission, genotype effects, appendiceal/tonsillar prevalence, and laboratory strain data on variant CJD (vCJD). Searches covered Jan 1, 1990, to Aug 7, 2025, with no language restrictions, using combinations of terms including “variant Creutzfeldt-Jakob disease” OR “vCJD” OR “nvCJD” OR “bovine spongiform encephalopathy” AND (“PRNP” OR “codon 129” OR “genotype” OR “incubation” OR “transfusion” OR “leukodepletion” OR “appendix” OR “tonsil” OR “prevalence” OR “modelling” OR “forecast”). We also hand-searched reference lists of key articles and national surveillance reports. Evidence comprised observational surveillance data (UK and international), cases of transfusion transmissions, large UK appendiceal screening studies, and prior projection models from the BSE era onwards. The evidence shows 232 recognised vCJD cases worldwide (178 UK) with the UK epidemic peaking around 2000 and no UK cases since a death in 2016; only one pathologically confirmed MV (non-MM) case has been described. Appendiceal surveys suggest abnormal prion protein in 1 in 2000–4000 samples. Earlier vCJD prediction models, notably from 2010, allowed for very large “tail” scenarios driven by assumptions about subclinical infection and secondary spread. Transfusion-linked clinical transmissions have been rare (three cases), with no events after universal leukodepletion (1999). Laboratory work indicates potential strain diversity, including a novel strain derived from the MV vCJD case in experimental systems. Overall risk of bias is moderate to high: surveillance under-ascertainment (especially with declining autopsy rates) and case rarity limit precision; appendiceal studies have sampling and interpretative uncertainties; and projections are structurally sensitive to unobserved parameters for non-MM genotypes.

**Added value of this study**

We provide an updated, genotype-stratified, cohort-based probabilistic model calibrated to the observed UK epidemic in MM individuals and explicitly incorporating competing mortality, likelihood weighting across non-MM parameter sets, and scenarios for missed historical diagnoses. In our base case, the single most likely outcome is zero additional cases (48% probability), with a low mean of 2.7 future cases; allowing for one or four missed cases modestly increases the mean to 3.0 and 4.9, respectively. A sensitivity analysis using a heavy-tailed (Cauchy) incubation distribution increases expected cases (6.6–17.9 across missed-case scenarios), quantifying the impact of tail assumptions. Even a deliberately pessimistic “worse-case” set of assumptions yields on the order of a few dozen cases (≈65), far below earlier high-end forecasts, and such a scenario would likely already have produced several recent cases, which have not been observed. We integrate these primary projections with empirical transfusion data to show that secondary transmission risk remains negligible under current safeguards. Together, these analyses reconcile historical pessimistic models with contemporary surveillance.

**Implications of all the available evidence**

A substantial second wave of vCJD in the UK now appears very unlikely. Policy can consider a rebalancing toward proportionate, durable measures: eg. universal leukodepletion, efforts to maintain case ascertainment, neuropathological confirmation, and laboratory capacity for strain characterization; and periodically revisit legacy controls (e.g., single-use instruments for medium-risk procedures) in light of the low projected risk and competing clinical priorities. Because non-MM cases may present atypically and autopsy rates have declined, strengthening pathways for post-mortem confirmation and modern molecular diagnostics in suspected CJD will preserve early-warning of a second wave. The quantitative forecasts support ongoing reviews of blood and plasma policies while emphasising that surveillance and specialist prion expertise should be sustained to detect rare late-onset cases or emergent strain phenomena promptly.

## Introduction

Variant Creutzfeldt–Jakob disease (vCJD) is a rare, fatal neurodegenerative disorder that emerged in the 1990s and was causally linked to the bovine spongiform encephalopathy (BSE) epizootic.^1^, ^2^, ^3^ It was first recognized in the United Kingdom in 1996, when investigators identified an unusual cluster of CJD cases in young patients and concluded these represented a new variant form of prion disease.^4^ BSE prions were soon confirmed as the source of vCJD through molecular and animal studies.^3^^,^^5^^,^^6^ In total, vCJD has accounted for 232 recognized cases worldwide as of 2024, with the vast majority (178 cases) occurring in the UK.^4^ Cases were reported in other countries (notably France with 28 cases, and smaller numbers in ten other nations), mostly attributed to exposure to BSE-contaminated beef during travel or importation. The vCJD epidemic peaked in the year 2000 and has declined steadily since then.^7^ The last known vCJD patient in the UK died in 2016 (symptom onset in 2014), and the most recent cases globally were reported in 2016 in Italy, and 2019 and 2021 in France, all three likely due to occupational exposure.^8^^,^^9^ Thanks to BSE control measures in the food chain, the risk of any new large outbreak from dietary exposure was essentially eliminated.

One striking feature of vCJD is its strong association with a genetic susceptibility factor in the prion protein gene (*PRNP*).^10^ Of tested patients with a definite diagnosis of vCJD (90% have been genotyped), all but one had the methionine/methionine (MM) genotype at codon 129 of the *PRNP*.^7^ This genotype is present in roughly 40% of European ancestries populations.^11^ The only pathologically-diagnosed exception to date is a single patient with the methionine/valine (MV) genotype, who died in 2016 in the UK,^12^ a further case was categorised as possible vCJD with an MV genotype, but without pathological confirmation, reported in 2011.^13^ No clinically suspected or confirmed cases have occurred in valine/valine homozygotes. The existence of an MV case after decades of latency supports the hypothesis that non-MM individuals can develop vCJD but may have longer incubation periods. Indeed, lessons from other prion diseases such as Kuru and iatrogenic CJD show that incubation periods over 30–50 years are possible in heterozygous individuals.^14^, ^15^, ^16^ This raises the concern that a “secondary wave” of vCJD cases could still appear among those with non-MM genotypes, even though the primary epidemic in MM individuals has run its course. Furthermore, vCJD has transmitted by blood transfusion resulting in clinical disease in three individuals, with evidence of transmission asymptomatically to two others, raising the possibility of a wave of secondary cases through iatrogenic human–human spread.^4^

A key uncertainty since the early years of the BSE crisis has been the number of people asymptomatically infected with vCJD prions. Abnormal prion protein in vCJD accumulates in peripheral lymphoreticular tissues like tonsil and appendix, and is readily detectable by immunohistology, unlike in other forms of human prion disease.^17^ Epidemiological screening studies of archived tissues have concluded that approximately 1 in 2000–4000 appendixes in the UK show accumulation of abnormal prion protein, in samples from individuals with no clinical signs of prion disease.^18^^,^^19^ This prevalence is far higher than the number of clinical vCJD cases and appears relatively uniform across birth cohorts. While these findings suggest that subclinical vCJD infections occurred in a subset of the population, their interpretation is challenging because abnormal appendixes were observed prior to 1980 before BSE was documented and in those born after 1996 when strict food safety measures had been introduced.^19^ It remains unclear what proportion of these putative carriers might eventually develop disease or be infectious to others. The discrepancy between the high presumed infection prevalence and the low number of clinical cases is a point of tension for public health, feeding into risk assessments for secondary transmission (e.g. via blood transfusion or surgical instruments), but perhaps resulting in disproportionate measures.

Given the above uncertainties, numerous modelling studies have been conducted to predict the future course of vCJD. Early models in the late 1990s produced a wide range of forecasts due to limited data. Some worse-case scenarios ran into many hundreds or thousands of eventual cases,^20^ the most recent paper in 2010 forecasting a median of 390 future cases with a 95% credible interval of 84–3000. The high-end outcome in that analysis depended on the assumption of many asymptomatic carriers contributing to secondary spread. In reality, no such secondary wave has yet appeared. Internationally, other countries' experiences have mirrored the UK's trajectory. France's cases peaked in the mid-2000s and then subsided, and no country has experienced a sustained resurgence of vCJD beyond the initial small outbreaks.

The UK's Advisory Committee for Dangerous Pathogens was commissioned to form a Subgroup of individuals (the authors) to update the modelling of vCJD risk in the UK and review the future risks. Our work builds on past epidemiological and modelling research and 12 years of case observation to more precisely quantify the likelihood and potential scale of any future vCJD cases. In particular, we incorporate genotype-specific parameters and explore scenario analyses (e.g. considering whether some cases of vCJD might have been missed) to account for remaining uncertainties. Finally, we discuss the potential policy implications of our projections.

## Methods

We developed a probabilistic model in Microsoft Excel to estimate the number of future vCJD cases in the UK arising from past exposure to BSE. The model follows the cohort of the full UK population during the period of exposure (primarily in the late 1980s and early 1990s) and projects deaths from vCJD over time, accounting for the incubation period distribution and competing risks. Individuals of all ages are considered to have been at risk of exposure. A key feature is the stratification by *PRNP* codon 129 genotype, given the stark difference in susceptibility between MM and non-MM individuals. The population was divided into two groups: 40% MM and 60% non-MM. The population was not stratified by sex or ethnicity. For each group, the model assumes a certain attack rate (the proportion of exposed individuals who eventually develop vCJD) and an incubation period distribution following a log-normal distribution parameterized by a mean and standard deviation. These parameters determine the timing and number of cases occurring, while standard life-table data were used to ensure that individuals could also die of other causes before manifesting vCJD, which is especially relevant for very long incubations. The choice of log-normal distribution for incubation period was chosen to reflect that incubation time must be non-negative but can potentially have a long tail.

**Fig. 1:**
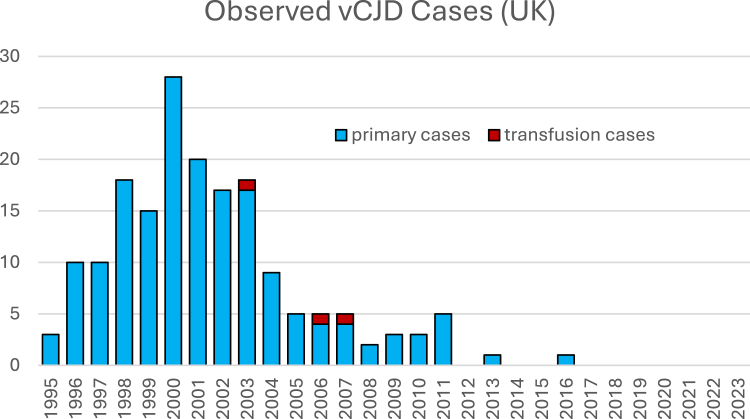
**Observed cases of definite and probable vCJD in the UK**. This figure shows the number of confirmed and probable vCJD cases in the UK over time, illustrating the rapid rise and subsequent decline of the epidemic following a peak in the early 2000s. The decline reflects the impact of public health interventions, including the removal of BSE-infected cattle from the food chain and stringent blood safety measures.

**Fig. 2:**
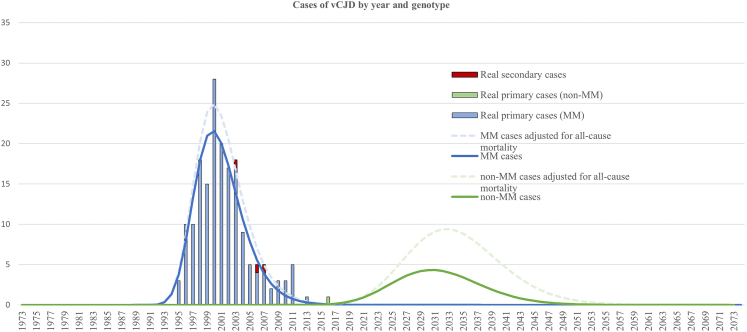
**Example projection**. An example projection based on the cohort model described in the Methods section, incorporating genotype-specific incubation periods and competing mortality risks. The figure demonstrates the potential future case counts under a plausible worse-case parameter set, highlighting a “second wave” vCJD risk in non-MM individuals over the coming decades. ∗Dashed lines indicate projections without, solid lines with, competing mortality risks. Note the dashed line for MM is very close to the solid line.

We calibrated the parameters for the MM model using the known UK epidemic curve in MM patients up to and including 2023. In practice, this meant finding values that reproduce the ∼174 MM cases from dietary exposure observed (Fig. 1), peaking around the year 2000 (example scenario and forecast, Fig. 2). The best–fit parameters yielded an MM attack rate on the order of 10^−5^, an average incubation time of 12.06 years, with a standard deviation of 3.41 years. These values are in line with prior analyses of the primary epidemic. Temporal validation showed that, although the model failed to predict the temporary slight rise in cases between 2008 and 2011, the key parameters (clinical attack rate, mean time till symptoms, and standard deviation of time till symptoms) did not vary significantly across validation scenarios (Table 1).

**Table 1:** Validation results.

Training years	Test years	Training:Test split	Fitted parameters			Cases in test years	
			CAR	Mean	Std dev	Actual	Prediction
Full data	NA	NA	0.05	12.06	3.41	NA	NA
1990–2006	2007–2023	12:7	0.05	11.76	2.97	18	6.8
1990–2008	2009–2023	14:5	0.05	11.82	3.05	12	2.9
1990–2010	2011–2023	16:3	0.05	11.95	3.28	6	1.6
2002–2023	1990–2001	12:7	0.06	11.46	3.92	104	148.3
2000–2023	1990–1999	14:5	0.07	10.81	3.50	56	138.2
1998–2023	1990–1997	16:3	0.05	12.12	3.39	23	25.8

**Fig. 3:**
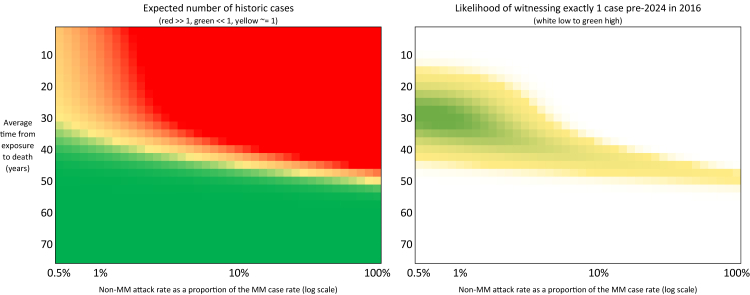
**Heat-maps of modelled historic non-MM cases and likelihood of model replicating the recorded cases**. Heatmaps illustrating the fit of various model parameter sets to the observed historical vCJD case data, including sensitivity to assumptions about incubation periods and attack rates. On the left, colours (red >1, green <1 and yellow ∼1) indicate expected numbers of model predicted historical cases. On the right, colours (white low to green high) indicate parameter combinations that are more likely to lead to the observation of a single non-MM case in 2016.

**Fig. 4:**
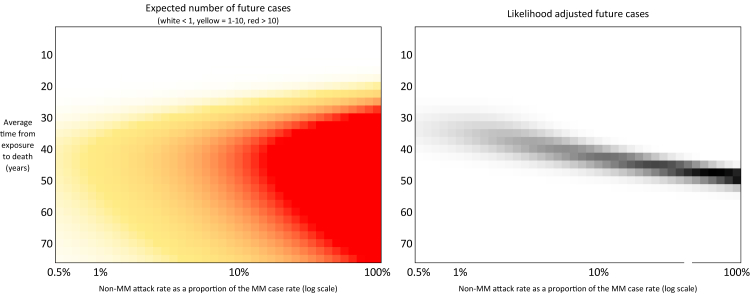
**Heat-maps of modelled future non-MM cases and likelihood adjusted future cases**. The heatmap on the left shows the expected number of future non-MM cases depending on parameter choice (coloured by white <1, yellow 1–10, and red >10). The heatmap on the right highlights the parameter choices that contribute the most to the expected number of future non-MM cases with darker being greater contribution. This is a trade off between plausibility and the number of cases predicted.

**Fig. 5:**
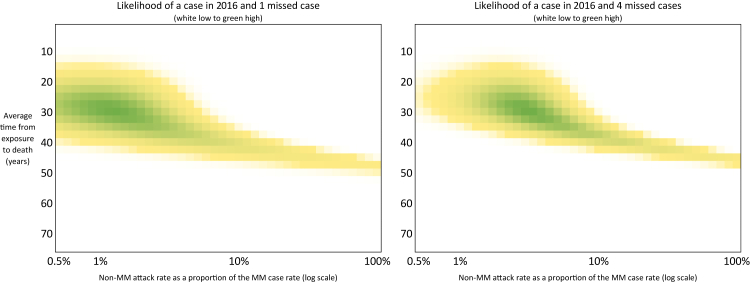
**Heat-maps of likelihood of model replicating historic recorded cases assuming some cases have been missed**. This figure presents the likelihood that various model parameter sets could account for the observed historic vCJD case data, under the assumption that some cases have been missed. It reflects the sensitivity of the model to under-ascertainment of past cases. Broadly, the models that predict 1 or 4 missed historical cases have a higher non-MM attack rate.

By contrast, the corresponding parameters for non-MM genotypes are not directly observable. We explored a wide plausible range for the non-MM attack rate, from very low (near-zero risk) up to as high as equal to the MM attack rate. Similarly, we examined incubation period assumptions for non-MM individuals that were generally longer than the MM mean, reflecting the possibility of extended latency (Fig. 3). Some scenarios considered average incubations of multiple decades for non-MM cases, consistent with the single MV case occurring over 25 years after likely exposure (Figs. 4 and 5).

For each combination of parameters, the model estimated the expected number of non-MM cases in each year, past and future. Assuming the true number of cases in each year follows a Poisson distribution with the mean matching this expectation, we derive the likelihood that each combination of parameters would lead to the observed number of cases in each year to date, i.e. the likelihood that the parameters would have led to exactly one non-MM case occurring in 2016 and no other non-MM cases. Other models such as zero-inflated models or negative binomial were considered but as overdispersion was not a significant issue for MM cases, a Poisson model was deemed most suitable.

For a given parameter set, P, the likelihood of those parameters leading to the observed case numbers is calculated as:

$$L_{P}≔Likelihood=\mathrm{Pr}\;(Exactly\;one\;historic\;case\;which\;occurs\;in\;2016)=\mu_{2016}*e^{-\mu_{2016}}*e^{-\left(\mu_{past}\;-\;\mu_{2016}\right)}$$

where $\mu_{2016}$ is the modelled number of non-MM cases in 2016 and $\mu_{past}$ is the modelled total number of historic non-MM cases. The single most likely parameter set is an incubation time of 30 years with non-MM attack rate being just 0.7% that of the MM attack rate. This corresponds to a peak lining up with the single non-MM case observed in 2016.

To determine the expected number of future cases we calculate a weighted average of the model predictions using this likelihood for the weighting.

$$Expected\;future\;cases=\frac{\sum_{P\in P}\mu_{future}*L_{P}}{\sum_{P\in P}L_{P}}$$

where P is the space of parameter combinations, $L_{P}$ is the likelihood weighting as defined above, and $\mu_{future}$ is the expected number of future cases for the given parameters.

To account for uncertainty in how many non-MM cases may have already occurred undetected, we look at two additional scenario variants. We consider one scenario in which there were two historic non-MM cases (the 2016 case and another hypothetical missed case that could have been in any year) and a scenario with four missed cases (these occurring in any years, and on top of the 2016 case). For both scenarios the model predicts the expected number of cases in each year, and we determine the likelihood of witnessing the specified number of historic cases by assuming the number of cases each year follows a Poisson distribution. Expert judgement from the Advisory Committee on Dangerous Pathogens was that more than four missed non-MM cases was highly implausible. Finally, although our model only addresses primary transmission (infection from BSE exposure), we also considered secondary transmission (human-to-human spread) in our analysis. We did not create a separate model for secondary cases because the number of such cases (3) has been very small. Instead, we reviewed existing case observation data on transfusion-transmitted vCJD and integrated those insights qualitatively with our primary case projections. This approach allows us to discuss the likelihood of any further secondary cases given the projected trend in primary cases.

The research used publicly available anonymised data only, so no research ethics or informed consent was needed.

### Role of the funding source

No specific funding for the project. No influence of the sponsors in study design; in the collection, analysis, and interpretation of data; in the writing of the report; and in the decision to submit the paper for publication.

## Results

In the base-case scenario (assuming all past vCJD cases have been identified), the model's projection indicates that zero further vCJD cases is the single most likely outcome with a 48% probability of no future cases (Table 2). There remains a significant chance (52%) of one or more cases occurring, but mostly at very low counts, for example, the probability of exactly one additional case is 25%, and the chance of two or three cases is under 15% combined. The expected value (mean) of the distribution is 2.7 future cases reflecting the weighting of a small probability of a few cases against the dominant zero-case scenario. Importantly, the upper tail of the prediction is very limited: the model estimates only around a 5% probability of seeing ten or more further cases in the coming decades. According to the model, the most likely timing for any additional vCJD outbreak has already passed—it suggests that the peak of cases among non-MM individuals likely occurred around the time of the lone 2016 case. While the model cannot completely rule out a second, later peak (e.g. in the 2030s), such an event is unlikely. In practical terms, a large second wave of vCJD in the UK appears highly unlikely under these assumptions. If any cases do occur in the future, they will likely be very few in number (most likely one).

**Table 2:** Key model parameters and outputs in different scenarios.

			Main model			Cauchy model		
			No missed non-MM cases	One missed non-MM case	Four missed non-MM cases	No missed non-MM cases	One missed non-MM case	Four missed non-MM cases
Inputs	Proportion of population that are MM		40%					
	Proportion of population that are non-MM		60%					
	Peak year of exposure		1989					
	Clinical attack rate, MM		0.05			0.02		
	Mean time till symptoms, MM (years)		12.06			11.37		
	Standard deviation of time till symptoms, MM (years)		3.41			2.44		
	Range of mean time till symptoms, non-MM (years)		2.5–75					
	Range of non-MM clinical attack rate relative to MM		0.5%–100%					
Outputs	Expected number of future cases		2.7	3.0	4.9	6.6	9.4	17.9
	Likelihood by number of future cases	zero	48%	50%	37%	0%	0%	0%
		1	25%	22%	21%	0%	0%	0%
		2	9%	9%	10%	22%	26%	22%
		3	5%	5%	6%	17%	9%	11%
		4	2%	2%	3%	11%	6%	4%
		5–9	6%	6%	10%	29%	22%	7%
		10–19	3%	3%	6%	16%	25%	15%
		20–29	3%	3%	6%	4%	13%	41%

This table summarizes the key model parameters and the expected number of cases under a range of model assumptions, including scenarios with no missed cases, one missed case, and four missed cases. Results for both our main analysis and our sensitivity analysis using a Cauchy model. Uncertainty is shown through presenting the likelihood of different numbers of cases. The Cauchy outputs include a fixed estimate of 1.9 future MM cases.

Introducing the possibility that some vCJD cases to date were not recognized produces only modest changes in the outlook. If one non-MM case was missed (i.e. a total of two such cases occurred historically, the one in 2016 and another at any time), the forecast mean rises slightly to ∼3.0 future cases (from 2.7), and the probability of zero further cases remains similar. This scenario is virtually indistinguishable from the base case in terms of projected risk. Even under a scenario where four cases were missed, occurring at any time, the model predicts only ∼4.9 future cases on average with a 37% chance of zero additional cases. The primary difference is a slight increase in the probability of a larger outbreak: with four missed cases, the chance of seeing 10 or more future cases rises to about 12% (versus ∼5% in the base case). In summary, unless a very substantial number of vCJD cases were somehow misdiagnosed—particularly in the very recent past—the forecast expected number of future cases remains very low. Only a scenario of multiple missed cases in the last few years would significantly raise the risk of a hidden upswing (by implying that the peak is actually underway now), and there is no positive evidence that such a cluster of misdiagnoses is occurring (see Discussion).

We also examined a hypothetical worse-case scenario to gauge the maximum plausible future burden of vCJD. In this scenario, non-MM individuals were assumed to have a relatively high susceptibility (attack rate approaching that of MM cases) and an extremely long average incubation period (∼45 years) (Fig. 2 illustrates the worse-case scenario). These conditions would imply that the peak incidence among non-MM (MV/VV) genotypes has not yet occurred but would be projected to occur around the early 2030s. Even under these pessimistic parameters, the model outputs were in the order of a few dozen future cases. Specifically, this worse-case scenario yielded 65 additional vCJD cases in the coming years, with an eventual peak of a few cases per year in the 2030s. Such an outcome would still be limited compared to the original epidemic. Additionally, this scenario would require that we have missed several non-MM cases. It also implies that if this worse-case were true, we would soon see evidence of it as the model forecasts on the order of 5–6 vCJD cases occurring between 2024 and 2026 in this scenario, which is not consistent with ongoing surveillance as of September 2025. Therefore, while tens of future cases cannot be absolutely ruled out, the conditions necessary for that scenario appear highly improbable, and it is expected that any significant future outbreak would reveal itself in the very near term.

To assess the robustness of our predictions, we conducted a sensitivity analysis by substituting the log-normal distribution for a Cauchy distribution, which has heavier tails and allows for a greater probability of outlier cases. This adjustment resulted in a significant increase in the projected number of future vCJD cases. Under the base-case assumption of no missed cases, the expected number of future cases rose from 2.7 to 6.6. If one case had been previously undiagnosed, the estimate increased from 3.0 to 9.4, and under the most pessimistic scenario where four historical cases were missed, the model projected an average of 17.9 additional cases, compared to the 4.9 cases estimated using the log-normal distribution. This highlights the sensitivity of long-term projections to the choice of incubation period distribution, particularly in the context of a rare but long-incubating disease. Notably, the increased expected case count in the sensitivity model was largely driven by scenarios in which the peak of a potential non-MM vCJD wave would occur between 2040 and 2060, at which point many at-risk individuals may have succumbed to other causes.

The Subgroup also considered the risk of secondary transmission (human-to-human spread, especially via blood transfusion). Empirical data show that there have been no transfusion-transmitted vCJD cases in the UK since 2006, and indeed only three such clinical cases were ever confirmed, all from non-leukodepleted red-cell transfusions received in the late 1990s. Our model results, which predict very few new primary infections, imply that opportunities for secondary transmission will be extremely scarce moving forward. We noted that secondary transmission has only definitively occurred from blood donors who became clinical cases of vCJD shortly after donation, with the implication that a low primary attack rate for non-MM donors will correlate with a low potential secondary transmission rate. In line with this, we did not find any scenario in which a large number of new secondary cases would be expected. Earlier projections^20^ that assumed a large subclinical infected population (and hence many infected blood donors) have not been borne out—those models greatly overestimated the number of secondary cases compared to what has actually occurred. In reality, all three known transfusion-related vCJD infections were linked to donors who developed vCJD symptoms within a couple of years after donation. Since leukodepletion of blood was introduced in 1999, no further transfusion transmissions have been observed. Given these facts, and the very low projection of future primary cases, the Subgroup considered that the most likely scenario is that no additional secondary transfusion-transmitted vCJD cases will occur.

## Discussion

Our findings suggest that the vCJD epidemic in the UK is unlikely to see a significant revival and will remain a single wave. In the most probable scenario, no further cases will occur, and even under pessimistic assumptions the projections are limited to a few dozen additional cases. Our modelling aligns with the epidemiological reality to date, that thankfully, vCJD cases have become exceedingly rare in the past decade, with no indications of a second wave emerging. Earlier models that entertained the possibility of large late-onset outbreaks now appear overly pessimistic, as the continued absence of cases has progressively tightened the upper bounds of risk. While uncertainty remains regarding the tail of the incubation period distribution, each passing year without new cases further reduces the plausibility of any substantial future epidemic. Despite this reassuring outlook, it is crucial to maintain vigilance and surveillance for vCJD for several reasons. Because the disease is invariably fatal, devastating for the individual patients and their families, and can have a long incubation, even a small number of cases would be of significant public health impact. A future wave of cases is not impossible, and would have very significant consequences.

Our modelling makes assumptions about prion biology which may not be correct. While our findings suggest that the vCJD epidemic has largely run its course, the nature of prion diseases leaves room for both developments that may be anticipated from analogies with other prion disorders, and others that remain unpredictable. The development of BSE and transmission to humans was largely unexpected, and vigilance is required against other unforeseen events that lead to prion disease from environmental sources. Experience of kuru and iatrogenic CJD (iCJD), demonstrate that codon 129 heterozygosity is associated with markedly prolonged incubation periods, sometimes exceeding 40 or even 50 years.^14^, ^15^, ^16^ These observations reinforce the plausibility of rare late-onset vCJD cases in MV individuals, and support our inclusion of long-tailed incubation distributions in the modelling. The recent report of iCJD with a 49-year incubation in an MV individual is consistent with this possibility,^16^ and further highlights the need for ongoing vigilance, particularly as atypical or mixed strain phenotypes may emerge in such long-incubated cases. Informed by expert consensus, we restricted the analysis to a maximum of four misdiagnosed cases of vCJD, as more extensive under-ascertainment was considered implausible given the strength of UK surveillance and the absence of confirmed cases since 2016 despite continued vigilance. While we cannot completely exclude the possibility of additional missed cases, our sensitivity analyses show that the impact on projected future case numbers remains modest unless under-ascertainment is substantial and recent.

Recent work by Zhang et al. (2025) has discovered that a previously unrecognized prion strain emerges from transmission experiments using brain tissue from the *PRNP* codon 129 MV vCJD patient, distinct from the classical vCJD strain.^21^ This novel strain was observed in humanized transgenic mice, exhibited enhanced transmissibility across different *PRNP* genotypes and a previously unseen neuropathological profile. These findings highlight the potential for prion strain evolution in the human population and the possibility that vCJD, rather than simply fading out, could persist in unexpected ways, whether through novel strain emergence which might mimic sporadic CJD, altered clinical presentations, or transmission routes not yet fully understood. Such possibilities reinforce the need for vigilance, ongoing case scrutiny and emphasize the importance of neuropathological examination and the laboratory facilities to study human prion strains in determining the true spectrum of prion diseases.

Another policy area needing careful consideration is blood safety. In response to vCJD, many countries implemented stringent blood donation restrictions to prevent secondary transmission. For instance, the UK has deferred donors with a history of blood transfusion, and the US barred donors who spent time in BSE-endemic countries. These measures have succeeded in preventing any known transfusion transmissions since 1999. Now, with vCJD incidence so low, some of these restrictions are being revisited. The U.S. Food and Drug Administration recently lifted the ban on blood donors who lived in the UK or Europe during the 1980s–90s, citing updated risk assessments.^22^^,^^23^ Likewise, in 2023 the UK ended its decades-old ban on using UK-sourced plasma for manufacturing medicinal products, and further changes are being considered.^23^^,^^24^ Such policy changes reflect growing confidence that the residual vCJD risk is very small, which our work supports. However, measures such as universal leukodepletion of blood components (implemented in 1999) should be maintained, as they would seem to have provided strong protection and have multiple benefits for non-prion related transfusion risks.^25^ The public's trust in the blood supply is paramount, and even a single transfusion-transmitted vCJD case in the future could undermine that trust.

Beyond blood transfusion, other public health measures established during the vCJD outbreak should be assessed in light of current evidence. One example is the use of single-use surgical instruments for procedures involving medium-risk tissues (e.g. tonsillectomy equipment), which was introduced in some jurisdictions to reduce the chances of iatrogenic prion transmission. Given the extremely low prevalence of vCJD now, policymakers could consider phasing out such costly measures, for instance, Scotland has debated the ongoing need for disposable tonsillectomy instruments. Any relaxation, however, must ensure a high quality of cleaning and sterilization for reusable surgical instruments. The recent (deaths in 2019 and 2021) French laboratory-acquired vCJD cases serve as a reminder of the potential for prion transmission by sharps injury, and that strict biosafety practices are non-negotiable and need to be regularly reviewed.^26^ Laboratories handling prions and hospitals performing neurosurgical procedures need to continue strict adherence to established occupational infection control guidelines to prevent accidental transmissions. Furthermore, the recent description of iatrogenic Alzheimer's disease and cerebral amyloid angiopathy emphasises a need to be mindful of other proteinopathies with routes of transmission that mirror classical prion diseases.^27^

While our projections focus on the risk of future vCJD cases, it is important to recognize that risk management decisions must consider a broader set of trade-offs. For example, the decision to continue using single-use surgical instruments to reduce potential prion transmission must be balanced against the potentially increased morbidity associated with procedures, such as tonsillectomies, where reusable instruments might have been safer. Similarly, the decision to resume the use of UK-sourced plasma for medicinal products reflects a broader risk-benefit analysis, balancing the very low residual vCJD risk against the clinical harms of reduced immunoglobulin availability for patients.

The only way to definitively diagnose vCJD is through neuropathological examination, yet the rate of autopsy in suspected CJD cases has dropped dramatically over time from about 70 to 80% two decades ago to below 10% in recent years.^7^ This decline in post-mortem examinations poses a risk that vCJD cases could go undetected or be misclassified as sporadic CJD, which we show has an influence on the forecasts. To guard against this, we recommend bolstering the resources and protocols for post-mortem examination in any unusual or suspect cases of neurodegenerative disease. Notably, the clinical presentation of the only MV genotype vCJD case (in 2016) more closely resembled sporadic CJD than classic vCJD, raising concern that future non-MM cases could be more difficult to recognize clinically.^12^ Strengthening the capacity of the UK surveillance to facilitate autopsies, for example, by providing funding, specialist support, and public education about the importance of autopsy for suspected prion cases, could be important. A return to higher autopsy rates would provide confidence that a nascent vCJD cluster would be recognized early, should one ever begin. In addition, maintaining diagnostic vigilance, including genetic testing for *PRNP* codon 129, and use of molecular diagnostics on biofluids taken in life in confirmed CJD cases, will help distinguish variant cases from sporadic CJD. Overall, support for specialist prion assessment is a way to ensure that the optimistic predictions hold true and no emerging threat is missed.

In conclusion, updated modelling suggests vCJD appears to be a diminishing but not a negligible future risk. The evidence and models concur that a large-scale second wave is very unlikely to occur in the UK. Nevertheless, prudence dictates that we do not declare the chapter completely closed until the cohort of people with dietary exposure to BSE currently aged 29+ has fully aged or definitive diagnostic tools for population studies confirm no persistent risk from asymptomatic infection. The potential policy implications of our findings are clear: maintenance of prion expertise and case scrutiny, including measures to reverse the decline in post-mortem confirmation, the ability to analyse human prions strains in atypical cases, and ongoing review of precautions in place in medical and blood transfusion practices. In practice, this means sustaining the clinical and laboratory infrastructure for CJD case investigation and being prepared to swiftly assess the implications of any new vCJD case or novel prion strain if one does arise. By doing so, public health authorities can ensure that the hard-won success in controlling BSE and vCJD is preserved, and that any future occurrences are promptly identified and understood. The story of vCJD stands as a testament to the importance of prescient and aggressive risk mitigation in the face of uncertainty and for now a lesson in how such measures, once effective, can be judiciously calibrated over time as the risk evolves.

## Contributors

Conceptualisation (SM TJE BR RK JM GM KS), data curation (BR JR RK), formal analysis (BR JR GM), data access and verification (BR JR), full access to data (BR JR SM) investigation (n/a), methodology (BR GM JR, with input from all authors), project administration (MU), resources (MU), software (BR JR), supervision (n/a), validation (BR JR), visualisation (BR JR), writing—original draft (SM) writing—review & editing (All authors) final decision to submit (All authors).

## Data sharing statement

The Excel model is available on Github: dhsc-govuk-internal/vCJD-forecast-model: Model forecasting the expected number of future cases of variant Creutzfeldt Jakob Disease. Readers can contact the corresponding author for data sharing.

## Declaration of interests

Nothing declared.
